# Supplementary material for: Spectroscopy of a tunable moiré system with a correlated and topological flat band
Source: Nat Commun. 2021 May 12;12:2732. doi: 10.1038/s41467-021-23031-0 (PMC8115081; doi:10.1038/s41467-021-23031-0)
Supplement: Supplementary file 1 — Supplementary Information [file 41467_2021_23031_MOESM1_ESM.pdf]

Supplementary Information for Spectroscopy of a Tunable Moiré System with a Correlated and Topological Flat Band

Xiaomeng Liu[1*], Cheng-Li Chiu[1*], Jong Yeon Lee[2], Gelareh Farahi[1], Kenji Watanabe[3], Takashi Taniguchi[4], Ashvin Vishwanath[2], Ali Yazdani[1‡]

[1]*Joesph Henry Laboratories and Department of Physics, Princeton University, Princeton, NJ 08544, USA*

[2]*Department of Physics, Harvard University, Cambridge, Massachusetts 02138, USA*

[3]*Research Center for Functional Materials, National Institute for Materials Science, 1-1 Namiki, Tsukuba 305-0044, Japan*

[4]*International Center for Materials Nanoarchitectonics, National Institute for Materials Science, 1-1 Namiki, Tsukuba 305-0044, Japan*

* These authors contributed equally to this work

‡ email: yazdani@princeton.edu

SUPPLEMENTARY FIGURES

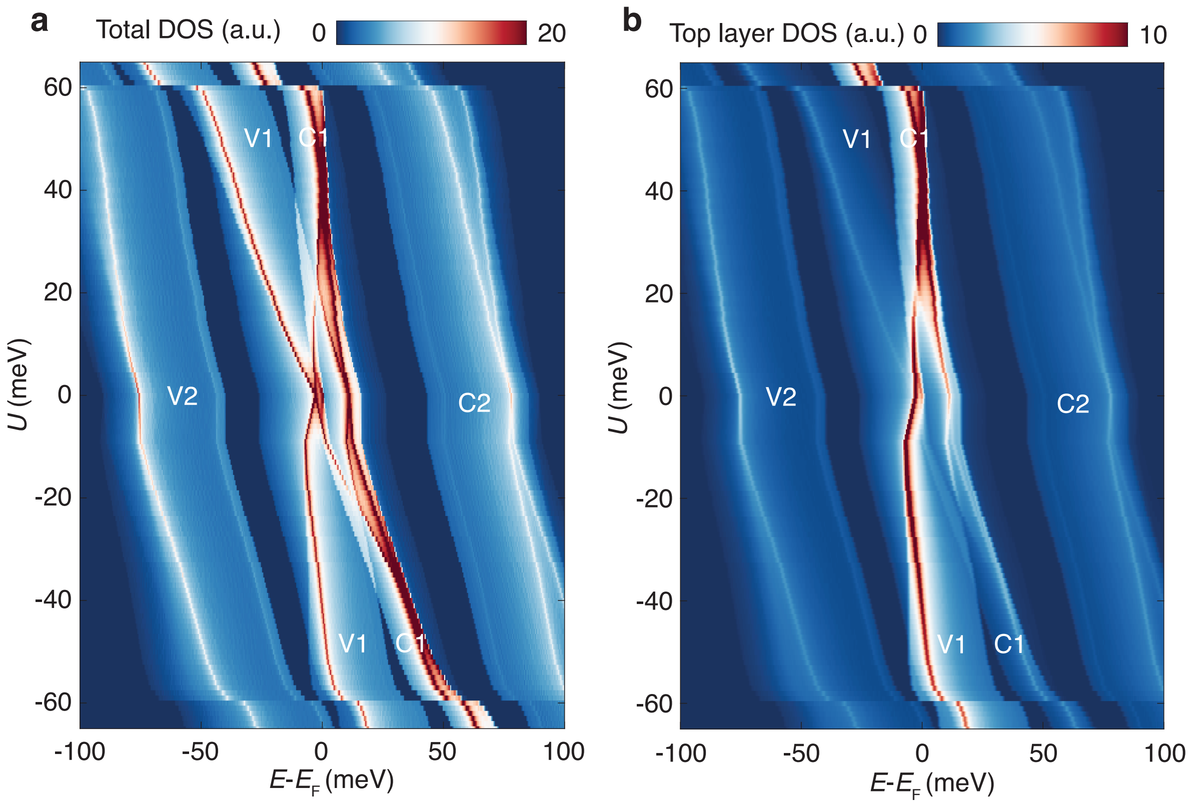

Supplementary Figure 1 | Calculated total density of state vs density of state on the top graphene layer. The total density of state (**a**) account for DOS on all four layers of graphene while panel (**b**) shows DOS on the top graphene layer.

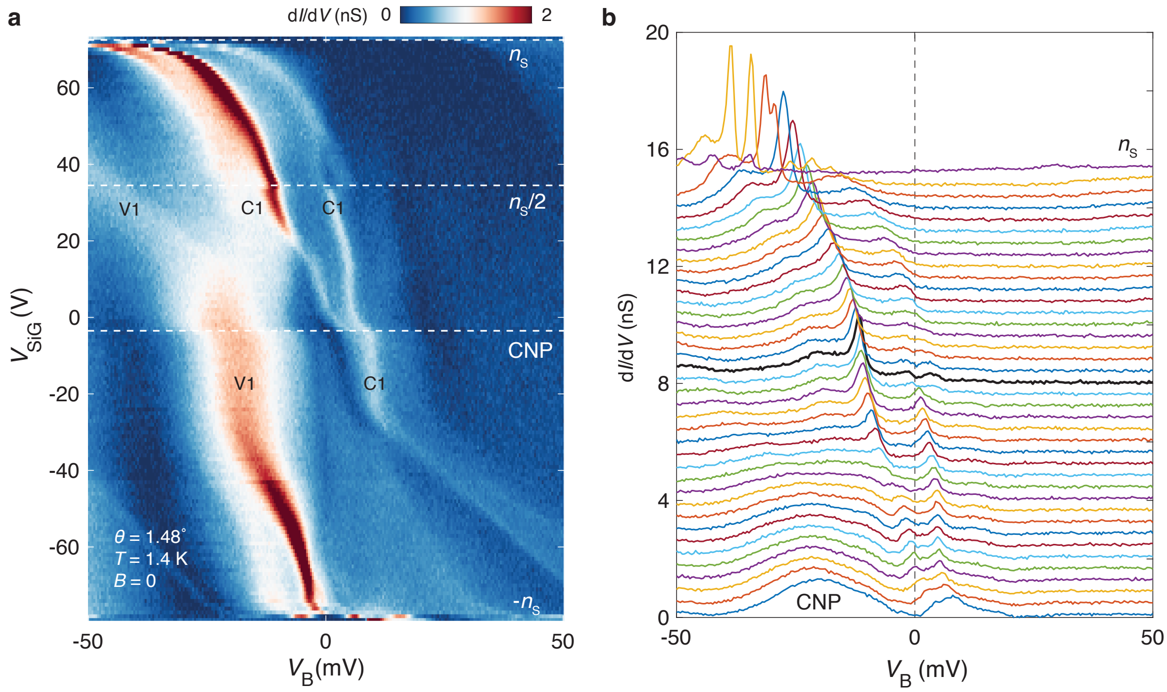

Supplementary Figure 2 | Gate-dependent spectra showing gaps at the Fermi level near half-filling of C1. a, STS measurements between -n_S and n_S, taken at ABBC stacking area (See Supplementary Fig. 2, area 4) , with V_{set} = -350 mV, I_{set} = 800 pA and V_{mod} = 0.5 mV. **b,** Line traces of the data presented in a. The curves are shifted vertically for clarity. The bottom curve is at charge neural point and the top curve is at $n_{S.}$ The trace at half filling is highlighted by the thicker black curve. We note a single peak split into two peaks at half filling.

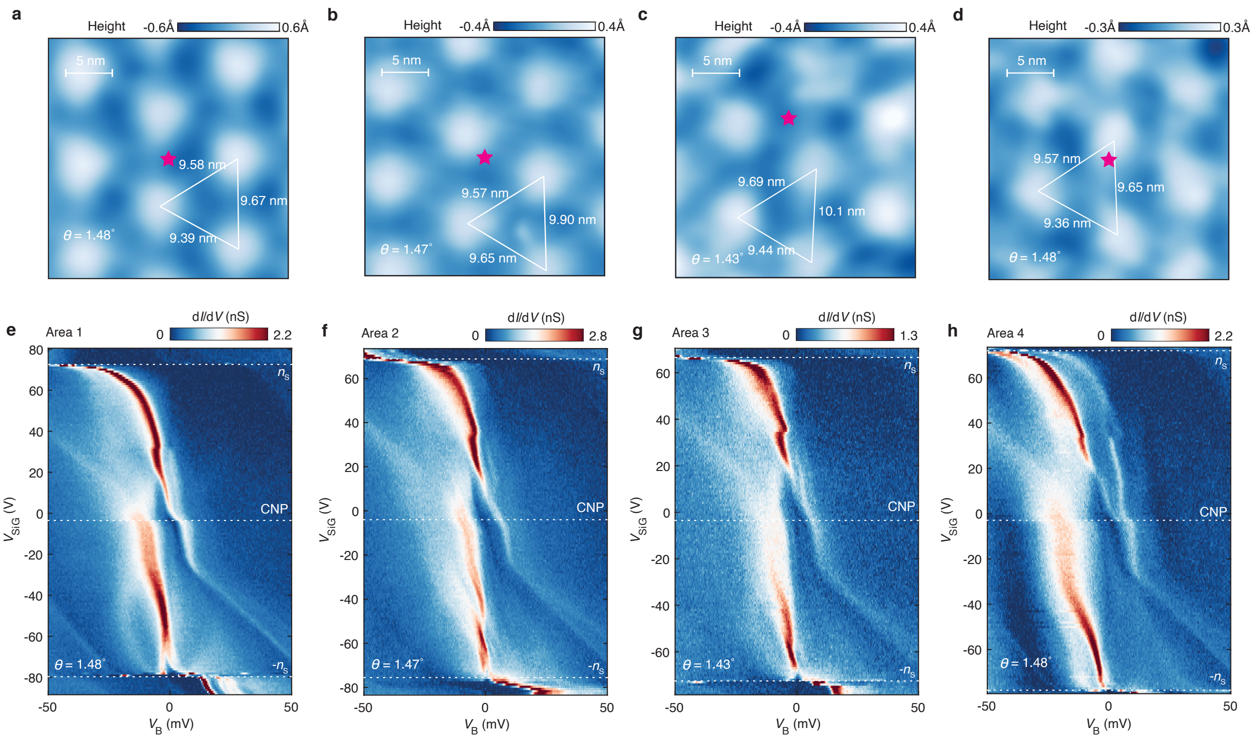

Supplementary Figure 3 | Topographs and gate-dependent STS measurements at various locations. a-d, STM topographs of TDBG taken at area 1, 2, 3 and 4 respectively. The bias set point V_{set} and current set point I_{set} are **a,** V_{set} = -150 mV, I_{set} = 200 pA. **b,** V_{set} = -400 mV, I_{set} = 40 pA. **c,** V_{set} = -400 mV, I_{set} = 50 pA. **d,** V_{set} = -400 mV, I_{set} = 70 pA. **e-h,** gate-dependent STS measurements taken at area 1, 2, 3 and 4 respectively. The red stars mark the location where the gate-dependent STS is taken. The white dashed lines mark the charge neutral point (CNP) and full filling of C1 (n_S) and V1 ($-n_S$) bands. All the data are taken with AC modulation V_{mod} = 0.5 mV. The bias set point V_{set} and current set point I_{set} are **e,** V_{set} = -400 mV, I_{set} = 800 pA. **f,** V_{set} = -300 mV, I_{set} = 800 pA. **g,** V_{set} = -400 mV, I_{set} = 800 pA. **h,** V_{set} = -400 mV, I_{set} = 800 pA.

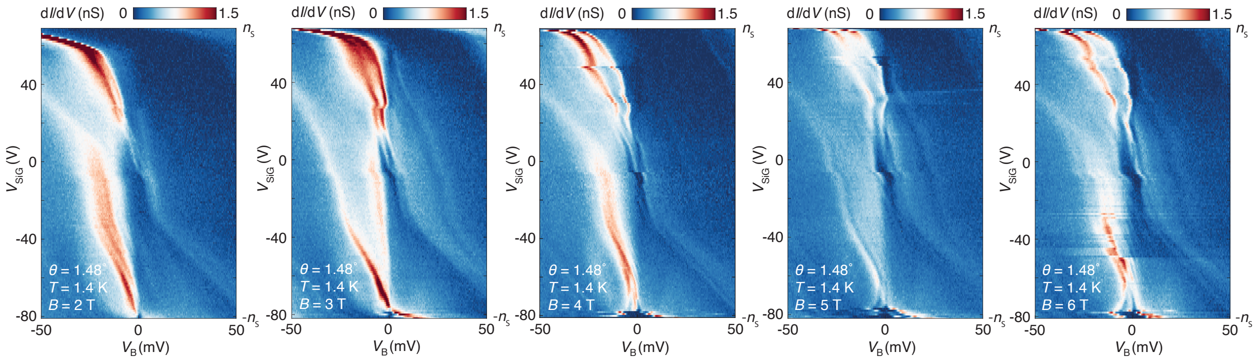

Supplementary Figure 4 | Gate-dependent STS measurements under various magnetic fields. STS measurements between $-n_s$ and n_s under five different magnetic fields. All data shown here are taken with voltage set point V_{set} = -400 mV, current set point I_{set} = 800 pA and AC modulation V_{mod} = 0.5 mV. The line-cuts and splitting shown in Fig. 5 are extracted from these data.

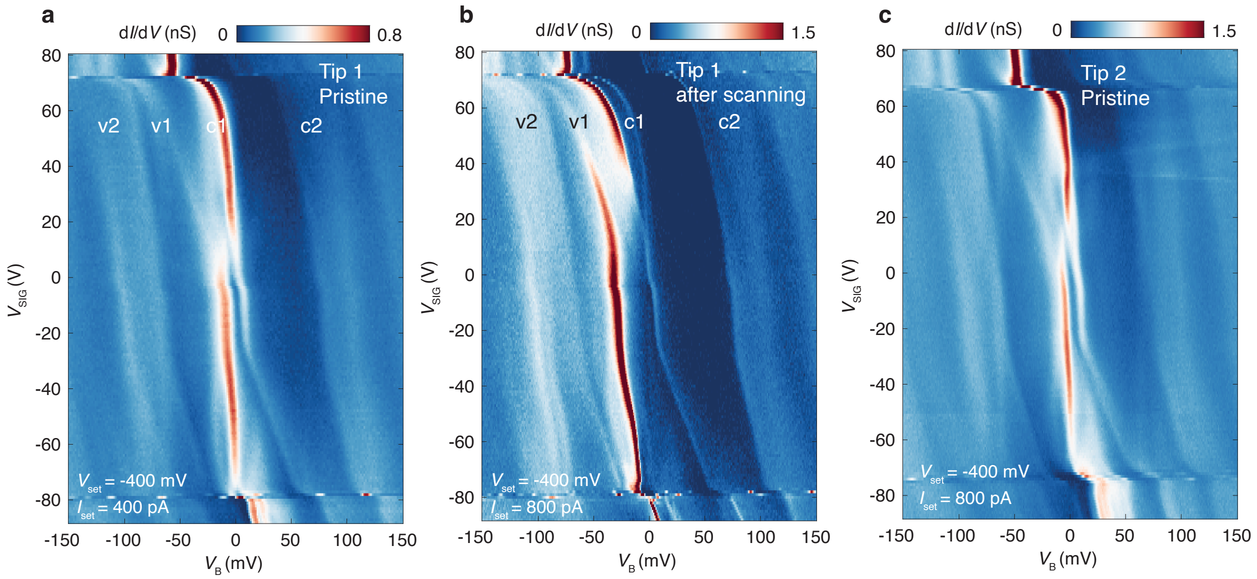

Supplementary Figure 5 | Effect of scanning on measured tunneling spectrum. Panel **(a)** and **(c)** show spectrums measured with freshly prepared tips without scanning. Panel **(b)** shows spectrum measured at the same spot as panel **(a)** after scanning images with the tip in panel **(a)**.

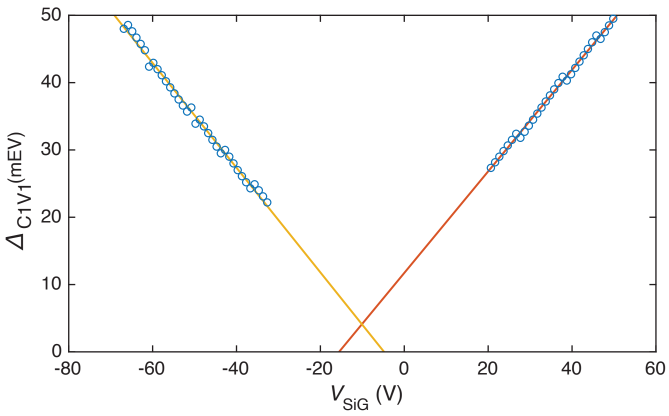

Supplementary Figure 6 | Estimate of build-in electric field. Gaps between C1 and V1 vHs are plotted as a function of back gate voltage. The two lines are linear fit to the gap at positive and negative electric fields. The estimate of back gate voltage at zero electric field can be read out from where the two lines intersect (-10 V). The build-in electric field is thus -10 V/300 nm*4/2 ~ 67 mV/nm, where 300 nm is the dielectric thickness and 4 is the dielectric constant.

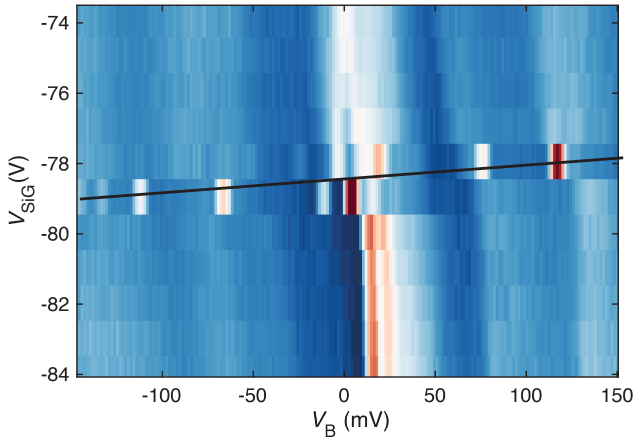

Supplementary Figure 7 | Tip gating effect. Solid black line provides a guide to full filling position as a function of bias voltage.

THEORETICAL MODELS

I. CONTINUUM MODEL CALCULATION

In this section, we elaborate how the density of states plot corresponding to the STM experiment can be calculated. We used the continuum model for TDBG as in the published work ([1]). The parameters used in our calculations are the following:

$$(\gamma_0, \gamma_1, \gamma_3, \gamma_4, \Delta, w_0, w_1) = (2700, 361, 283, 138, 15, 88, 110) \text{ meV}. \tag{1}$$

For given model parameters, we calculated band structures for different set of twist angles θ and potential difference between layers U (which is spread out among four graphene layers as (-U/2, -U/6, U/6, U/2)). The density of state on the top graphene layer is extracted by considering the wavefunction distribution of the top layer. Each eigenstate $\Psi_{n,\tau,\sigma}(\boldsymbol{k})$ is labelled by the band index n, valley index τ, spin index σ, and momenta $\boldsymbol{k}$, consisting of eight components – four layers, and two sublattice sites A and B for each layer. To obtain the density of states in the top-most layer (for each valley and spin), we evaluated the following:

$$\rho^{\text{top}}_{\tau,\sigma}(E)\Delta E = \sum_{E_{\boldsymbol{k}} \in (E, E+\Delta E)} \left(\left| \Psi^{A1}_{n,\tau,\sigma}(\boldsymbol{k}) \right|^2 + \left| \Psi^{B1}_{n,\tau,\sigma}(\boldsymbol{k}) \right|^2 \right) \tag{2}$$

where ΔE is the energy resolution. To compare with the STM experiment in Fig. 3, we must shift the calculated density of state in energy so that it is plotted as a function of the bias voltage V_B, which is equivalent to the energy relative to the Fermi energy $E - E_F$. Note that the Fermi energy is determined by the electron density n_e and the band structure. Therefore, we have to know the electron density n_e at each value of U.

In the experiment, the silicon gate voltage tunes both the carrier density and electric field simultaneously. It is fair to assume that both quantities change linearly in the gate voltage. Based on the experimental observation of the gap closings between the first and second valence bands, we estimated the correspondence between the continuum model parameter U and experimental gate voltage V_{SiG}, which in turn is proportional to the electron density n_e. As a result, in the calculations, we use the following quantitative relation between the electron density n and U:

$$n_e = n_s \cdot \frac{U}{60 \text{ meV}}, \tag{3}$$

where n_s corresponds to the full-filling electron density, which is four electrons per Moire unit cell.

With a given electron density n_e and band structure at certain U, it is straightforward to evaluate the Fermi energy. Here we used the mesh size of 80×80 in the momentum space.

II. REAL SPACE DENSITY OF STATES

In the tight-binding model, there is no real space distribution of real space density for a particular state. However, if each unit cell contains multiple sites, there can be a non-uniform distribution of electrons within the unit cell. Similar thing happens for the continuum model where each wavefunction is the linear combination of Fourier components which differ by Moire reciprocal lattice vectors. In numerics, each eigenvector corresponds to the following vector

$$\Psi(\boldsymbol{k}) = (u_{\boldsymbol{k}}(\boldsymbol{G}_1), u_{\boldsymbol{k}}(\boldsymbol{G}_2), u_{\boldsymbol{k}}(\boldsymbol{G}_3), ..., u_{\boldsymbol{k}}(\boldsymbol{G}_i), ...), \tag{4}$$

where G_i is the set of Moire reciprocal lattice vectors. In practice, we truncate at certain point and keep only a finite number of Fourier components. Its spatial distribution within the Moire unit cell is simply calculated as the following:

$$\rho_{\boldsymbol{k}}(\boldsymbol{r}) = |\Psi_{\boldsymbol{k}}(\boldsymbol{r})|^2 = \left| \sum_{\boldsymbol{G}} e^{i(\boldsymbol{k}+\boldsymbol{G})\cdot \boldsymbol{r}} u_{\boldsymbol{k}}(\boldsymbol{G}) \right|^2. \tag{5}$$

Finally, the real space density plot can be summed over the energy window we are interested in.

III. CHERN NUMBER AND g-FACTOR

In the main text, we remarked that the observed large g-factor is related to the band carrying non-zero Chern number. This can be understood by examining how they are defined. A Chern number is defined as the integration of the Berry curvature, which is defined as the following:

$$C_n = \frac{1}{2\pi} \int d^2k\, \Omega_n(\boldsymbol{k}), \qquad \Omega_n(\boldsymbol{k}) = i \sum_{m \neq n} \frac{\langle n | \partial_{k_x} H_{\boldsymbol{k}} | m \rangle - \text{h.c.}}{(E_n - E_m)^2}, \tag{6}$$

where $\Omega_n(\boldsymbol{k})$ is the berry curvature of n-th band at momentum $\boldsymbol{k}$. This definition is consistent with the definition where the electron's Landau level carries $C = +1$. Note that $\sigma_{yx} \propto C$ in this case. The formula for berry curvature is similar to the orbital g-factor defined as the following [1]:

$$g_n(\boldsymbol{k}) = \frac{2m_e}{\hbar} i \sum_{m \neq n} \frac{\langle n | \partial_{k_x} H_{\boldsymbol{k}} | m \rangle - \text{h.c.}}{E_n - E_m}, \tag{7}$$

where m_e is the free electron mass. The main difference between $\Omega_n(\boldsymbol{k})$ and $g_n(\boldsymbol{k})$ is the denominator.

For simplicity, consider a two-band model with $E_1(\boldsymbol{k}) > E_2(\boldsymbol{k})$. There, it is easy to notice that $\Omega_1(\boldsymbol{k}) = -\Omega_2(\boldsymbol{k})$. Furthermore, we can notice that

$$g_1(\boldsymbol{k}) = g_2(\boldsymbol{k}) = \frac{2m_e}{\hbar}(E_1 - E_2) \cdot \Omega_1(\boldsymbol{k}). \tag{8}$$

Therefore, we can deduce the relation between averaged g-factor and Chern number as the following

$$g_1^{\text{ave}} = g_2^{\text{ave}} \sim \frac{2m_e}{\hbar}(E_1 - E_2) \cdot \frac{2\pi C_1}{A_{\text{BZ}}} \tag{9}$$

where A_{BZ} is the area of the Brillouin zone. Of course, this would not hold if each band is so dispersive that the gap $E_1(\boldsymbol{k}) - E_2(\boldsymbol{k})$ fluctuates significantly, which gives a correction proportional to the manitude of the fluctuation. However, assuming two bands are well separated and relatively flat, one can associate Chern number and g-factor averaged over the Brillouin zone.

In twisted materials, know that g-factors satisfy the following relationship between opposite valleys:

$$g_{n,+}(\boldsymbol{k}) = -g_{n,-}(-\boldsymbol{k}) \quad \Rightarrow \quad g_{n,+}^{\text{ave}} = -g_{n,-}^{\text{ave}}. \tag{10}$$

This implies that under the strong perpendicular magnetic field, we would observe the splitting of van-Hove singularities originated from different valleys. In our TDBG system, we notice that the first conduction and valence bands are well isolated from the other bands. Over the range of U values we calculated, the direct gap between first and second conduction (valence) bands is factor of 2 to 4 times larger than the direct gap between first conduction and valence bands. Therefore, we can associate the observation of large g-factor and the band carryring non-zero Chern number.

[1] J. Y. Lee, E. Khalaf, S. Liu, X. Liu, Z. Hao, P. Kim, and A. Vishwanath, Theory of correlated insulating behaviour and spin-triplet superconductivity in twisted double bilayer graphene, Nature Communications **10**, 5333 (2019).
